# Hexaaqua­magnesium(II) bis­[4-(3-pyrid­yl)pyrimidine-2-sulfonate] tetra­hydrate

**DOI:** 10.1107/S160053680904344X

**Published:** 2009-10-28

**Authors:** Yan-Ping Wang, Jian Li, Hua-Ze Dong

**Affiliations:** a Department of Chemistry and Chemical Engineering, Hefei Normal University, Hefei 230061, People’s Republic of China

## Abstract

The asymmetric unit of the title compound, [Mg(H_2_O)_6_](C_9_H_6_N_3_O_3_S)_2_·4H_2_O, contains half of a centrosymmetric cation, one 4-(3-pyrid­yl)pyrimidin-2-sulfonate anion and two solvent water mol­ecules. Inter­molecular O—H⋯O and O—H⋯N hydrogen bonds link the cations, anions and water mol­ecules into a three-dimensional supra­molecular structure. The crystal packing also exhibits inter­molecular π–π inter­actions between the aromatic rings of the anions with a centroid–centroid distance of 3.604 (2) Å.

## Related literature

For coordination complexes with pyridin-2-sulfonate ligands, see: Kimura *et al.* (1999 ▶); Lobana *et al.* (2004 ▶). For coordination complexes with 4-(pyridin-yl)pyrimidin-2-sulfonate, see: Zhu *et al.* (2007 ▶); Fang *et al.* (2009 ▶).
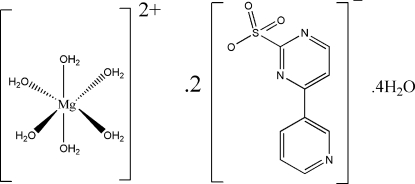


## Experimental

### 

#### Crystal data

[Mg(H_2_O)_6_](C_9_H_6_N_3_O_3_S)_2_·4H_2_O
*M* *_r_* = 676.95
Monoclinic, 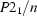

*a* = 6.9835 (2) Å
*b* = 13.3600 (3) Å
*c* = 16.2565 (4) Å
β = 98.7240 (10)°
*V* = 1499.18 (7) Å^3^
*Z* = 2
Mo *K*α radiation
μ = 0.28 mm^−1^
*T* = 291 K
0.30 × 0.15 × 0.12 mm

#### Data collection

Bruker SMART CCD area-detector diffractometer
Absorption correction: multi-scan (*SADABS*; Bruker, 2000 ▶) *T*_min_ = 0.917, *T*_max_ = 0.966
14712 measured reflections
3438 independent reflections
2848 reflections with *I* > 2σ(*I*)
*R*_int_ = 0.025

#### Refinement

*R*[*F* ^2^ > 2σ(*F* ^2^)] = 0.038
*wR*(*F* ^2^) = 0.102
*S* = 1.04
3438 reflections
236 parameters
7 restraints
H atoms treated by a mixture of independent and constrained refinement
Δρ_max_ = 0.23 e Å^−3^
Δρ_min_ = −0.40 e Å^−3^

### 

Data collection: *SMART* (Bruker, 2000 ▶); cell refinement: *SAINT* (Bruker, 2000 ▶); data reduction: *SAINT*; program(s) used to solve structure: *SHELXTL* (Sheldrick, 2008 ▶); program(s) used to refine structure: *SHELXTL*; molecular graphics: *SHELXTL*; software used to prepare material for publication: *SHELXTL*.

## Supplementary Material

Crystal structure: contains datablocks I, global. DOI: 10.1107/S160053680904344X/cv2632sup1.cif

Structure factors: contains datablocks I. DOI: 10.1107/S160053680904344X/cv2632Isup2.hkl

Additional supplementary materials:  crystallographic information; 3D view; checkCIF report

## Figures and Tables

**Table 1:** Hydrogen-bond geometry (Å, °)

*D*—H⋯*A*	*D*—H	H⋯*A*	*D*⋯*A*	*D*—H⋯*A*
O4—H4*A*⋯O1^i^	0.83 (2)	1.94 (2)	2.7650 (19)	180 (3)
O4—H4*B*⋯O2	0.86 (2)	1.89 (2)	2.7475 (19)	176 (2)
O5—H5*B*⋯O3^i^	0.83 (2)	2.04 (2)	2.8705 (19)	177.0 (18)
O5—H5*A*⋯O8^ii^	0.86 (2)	1.91 (2)	2.755 (3)	167 (3)
O6—H6*B*⋯O3^iii^	0.83 (2)	2.03 (3)	2.8601 (19)	178 (2)
O6—H6*A*⋯N3^iv^	0.85 (3)	1.92 (3)	2.763 (2)	173 (2)
O7—H7*B*⋯O3^v^	0.84 (3)	2.51 (3)	3.110 (2)	130 (3)
O7—H7*B*⋯N2^v^	0.84 (3)	2.21 (3)	2.984 (2)	154 (3)
O7—H7*A*⋯O2	0.84 (3)	2.12 (3)	2.923 (2)	161 (3)
O8—H8*B*⋯O1^vi^	0.85 (4)	2.43 (4)	3.064 (2)	132 (4)
O8—H8*A*⋯O7^iii^	0.85 (3)	1.93 (3)	2.769 (3)	170 (3)

Symmetry codes: (i) 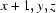; (ii) 
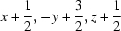
; (iii) 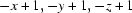; (iv) 
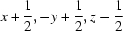
; (v) 
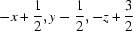
; (vi) 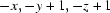.

**Fig. 1.**
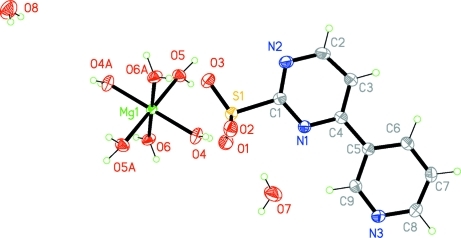
A portion of the crystal structure of (I) showing 30% probability displacement ellipsoids and the atom-numbering scheme [symmetry code: (A) -1 - x, 1 - y, 1 - z].

## supplementary crystallographic information

### Comment

The rational design and synthesis of coordination complexes derived from heterocyclic sulfonate ligands have been of increasing interest recently in chemical research (Kimura *et al.*, 1999; Lobana *et al.*, 2004). In our previous work (Zhu *et al.*, 2007; Fang *et al.*, 2009), we have also studied transition metal coordination complexes with the heterocyclic sulfonate ligands, namely 4-(pyridin-2-yl)pyrimidin-2-sulfonate and 4-(pyridin-4-yl)pyrimidin-2-sulfonate. Herein, we report the magnesium(II) coordination complex with its analog, *viz.* 4-(pyridin-3-yl)pyrimidin-2-sulfonate.

The asymmetric unit of the title compound (Fig. 1) consists of a 4-(3-pyridyl)pyrimidin-2-sulfonate anion, one half of an [Mg(H_2_O)_6_]^2+^ cation and two free water molecules. The averaged Mg—O coordinating bond length is 2.0664 (13) Å. In the crystal structure, intermolecular O—H···O and O—H···N hydrogen bonds (Table 1) link cations, anions and crystalline water molecules into three-dimensinal network. The crystal packing exhibits also intermolecular π—π interactions between the aromatic rings of the anions with the centroid-centroid distance of 3.604 (2) Å.

### Experimental

All solvents and chemicals were of analytical grade and were used without further purification. 4-(3-Pyridyl)pyrimidin-2-sulfonate (L) was prepared by similar procedure reported in the literature (Zhu *et al.*, 2007; Fang *et al.*, 2009). For the synthesis of title compoud, a solution of L (0.1 mmol), MgSO_4 _(0.1 mmol) in 30 ml methanol was stirred for 1 h at room temperature. After filtration, the mother liguid was stood for one week to give the colourless crystals suitable for X-ray diffraction annalysis.

### Refinement

C-bound H atoms were placed in geometrically idealized positions (C—H 0.93 Å) and treated as riding on their parent atoms , with *U*_iso_(H)=1.2*U*_eq_(C). O-bound H atoms were located on a difference map and refined isotropically with the bond restraint O—H = 0.84 (2) Å.

### Crystal data

**Table:**

[Mg(H_2_O)_6_](C_9_H_6_N_3_O_3_S)_2_·4H_2_O	*F*(000) = 708
*M**_r_* = 676.95	*D*_x_ = 1.500 Mg m^−^^3^
Monoclinic, *P*2_1_/*n*	Mo *K*α radiation, λ = 0.71073 Å
Hall symbol: -P 2yn	Cell parameters from 15164 reflections
*a* = 6.9835 (2) Å	θ = 2.0–27.5°
*b* = 13.3600 (3) Å	µ = 0.28 mm^−^^1^
*c* = 16.2565 (4) Å	*T* = 291 K
β = 98.724 (1)°	Block, colourless
*V* = 1499.18 (7) Å^3^	0.30 × 0.15 × 0.12 mm
*Z* = 2	

### Data collection

**Table:**

Bruker SMART CCD area-detector diffractometer	3438 independent reflections
Radiation source: fine-focus sealed tube	2848 reflections with *I* > 2σ(*I*)
graphite	*R*_int_ = 0.025
φ and ω scans	θ_max_ = 27.5°, θ_min_ = 2.0°
Absorption correction: multi-scan (*SADABS*; Bruker, 2000)	*h* = −9→9
*T*_min_ = 0.917, *T*_max_ = 0.966	*k* = −17→15
14712 measured reflections	*l* = −19→21

### Refinement

**Table:**

Refinement on *F*^2^	Primary atom site location: structure-invariant direct methods
Least-squares matrix: full	Secondary atom site location: difference Fourier map
*R*[*F*^2^ > 2σ(*F*^2^)] = 0.038	Hydrogen site location: inferred from neighbouring sites
*wR*(*F*^2^) = 0.102	H atoms treated by a mixture of independent and constrained refinement
*S* = 1.04	*w* = 1/[σ^2^(*F*_o_^2^) + (0.0509*P*)^2^ + 0.508*P*] where *P* = (*F*_o_^2^ + 2*F*_c_^2^)/3
3438 reflections	(Δ/σ)_max_ = 0.001
236 parameters	Δρ_max_ = 0.23 e Å^−^^3^
7 restraints	Δρ_min_ = −0.40 e Å^−^^3^

### Special details

**Table:**

Experimental. The structure was solved by direct methods (Bruker, 2000) and successive difference Fourier syntheses.
Geometry. All e.s.d.'s (except the e.s.d. in the dihedral angle between two l.s. planes) are estimated using the full covariance matrix. The cell e.s.d.'s are taken into account individually in the estimation of e.s.d.'s in distances, angles and torsion angles; correlations between e.s.d.'s in cell parameters are only used when they are defined by crystal symmetry. An approximate (isotropic) treatment of cell e.s.d.'s is used for estimating e.s.d.'s involving l.s. planes.
Refinement. Refinement of *F*^2^ against ALL reflections. The weighted *R*-factor *wR* and goodness of fit *S* are based on *F*^2^, conventional *R*-factors *R* are based on *F*, with *F* set to zero for negative *F*^2^. The threshold expression of *F*^2^ > σ(*F*^2^) is used only for calculating *R*-factors(gt) *etc*. and is not relevant to the choice of reflections for refinement. *R*-factors based on *F*^2^ are statistically about twice as large as those based on *F*, and *R*- factors based on ALL data will be even larger.

### Fractional atomic coordinates and isotropic or equivalent isotropic displacement parameters (Å^2^)

**Table:**

	*x*	*y*	*z*	*U*_iso_*/*U*_eq_	
Mg1	0.5000	0.5000	0.5000	0.03117 (19)	
S1	0.05652 (6)	0.48762 (3)	0.71033 (2)	0.03373 (13)	
N1	0.1709 (2)	0.47749 (10)	0.87143 (9)	0.0339 (3)	
O1	−0.12580 (18)	0.43964 (10)	0.71840 (8)	0.0441 (3)	
O2	0.20815 (19)	0.41651 (10)	0.70020 (8)	0.0484 (3)	
N3	0.3137 (2)	0.26720 (11)	1.05433 (9)	0.0425 (4)	
N2	0.1324 (2)	0.64241 (10)	0.81545 (9)	0.0416 (3)	
C4	0.2220 (2)	0.51444 (12)	0.94866 (10)	0.0320 (3)	
C1	0.1302 (2)	0.54365 (12)	0.81052 (10)	0.0324 (3)	
O3	0.03921 (19)	0.56801 (9)	0.64992 (8)	0.0443 (3)	
C9	0.2743 (3)	0.33887 (12)	0.99732 (11)	0.0380 (4)	
H9	0.2487	0.3200	0.9417	0.046*	
C5	0.2691 (2)	0.44024 (12)	1.01627 (10)	0.0319 (3)	
C6	0.3095 (3)	0.46696 (14)	1.09979 (10)	0.0411 (4)	
H6	0.3084	0.5339	1.1155	0.049*	
C8	0.3510 (3)	0.29500 (14)	1.13397 (11)	0.0448 (4)	
H8	0.3782	0.2456	1.1744	0.054*	
C3	0.2265 (3)	0.61758 (13)	0.96124 (11)	0.0412 (4)	
H3	0.2593	0.6445	1.0142	0.049*	
C7	0.3512 (3)	0.39317 (14)	1.15898 (11)	0.0466 (4)	
H7	0.3792	0.4096	1.2151	0.056*	
C2	0.1811 (3)	0.67811 (13)	0.89291 (12)	0.0465 (4)	
H2	0.1843	0.7471	0.9006	0.056*	
O7	0.4762 (3)	0.29673 (14)	0.81603 (13)	0.0812 (6)	
H7A	0.407 (5)	0.342 (2)	0.791 (2)	0.137 (15)*	
H7B	0.438 (5)	0.2425 (19)	0.793 (2)	0.133 (14)*	
O6	0.7440 (2)	0.42088 (10)	0.48561 (9)	0.0445 (3)	
O4	0.5193 (2)	0.43705 (11)	0.61644 (8)	0.0477 (3)	
O5	0.6808 (2)	0.61790 (10)	0.54696 (9)	0.0467 (3)	
O8	0.1251 (4)	0.72373 (16)	0.14928 (13)	0.0869 (6)	
H6A	0.773 (3)	0.3654 (19)	0.5094 (15)	0.064 (7)*	
H6B	0.804 (4)	0.425 (2)	0.4457 (14)	0.081 (8)*	
H4B	0.422 (3)	0.4341 (17)	0.6427 (14)	0.056 (6)*	
H5A	0.647 (4)	0.6623 (18)	0.5805 (15)	0.088 (9)*	
H5B	0.786 (3)	0.602 (2)	0.5755 (16)	0.085 (9)*	
H4A	0.626 (3)	0.438 (2)	0.6468 (15)	0.074 (8)*	
H8A	0.245 (4)	0.717 (2)	0.1660 (17)	0.072 (9)*	
H8B	0.055 (6)	0.681 (3)	0.169 (3)	0.168 (19)*	

### Atomic displacement parameters (Å^2^)

**Table:**

	*U*^11^	*U*^22^	*U*^33^	*U*^12^	*U*^13^	*U*^23^
Mg1	0.0328 (4)	0.0308 (4)	0.0295 (4)	0.0031 (3)	0.0035 (3)	0.0025 (3)
S1	0.0337 (2)	0.0357 (2)	0.0314 (2)	−0.00007 (16)	0.00366 (16)	0.00238 (16)
N1	0.0345 (7)	0.0317 (7)	0.0342 (7)	−0.0004 (6)	0.0008 (6)	0.0002 (6)
O1	0.0399 (7)	0.0520 (7)	0.0390 (7)	−0.0115 (6)	0.0012 (5)	−0.0002 (6)
O2	0.0504 (8)	0.0518 (7)	0.0445 (7)	0.0138 (6)	0.0115 (6)	0.0012 (6)
N3	0.0540 (9)	0.0327 (7)	0.0396 (8)	−0.0044 (7)	0.0038 (7)	0.0016 (6)
N2	0.0472 (9)	0.0315 (7)	0.0440 (8)	−0.0003 (6)	−0.0003 (7)	0.0036 (6)
C4	0.0270 (8)	0.0330 (8)	0.0357 (8)	−0.0004 (6)	0.0034 (6)	−0.0023 (7)
C1	0.0271 (8)	0.0328 (8)	0.0364 (8)	−0.0010 (6)	0.0018 (6)	0.0027 (7)
O3	0.0493 (7)	0.0459 (7)	0.0370 (7)	0.0006 (6)	0.0044 (5)	0.0092 (5)
C9	0.0433 (10)	0.0354 (8)	0.0337 (8)	−0.0031 (7)	0.0011 (7)	−0.0024 (7)
C5	0.0287 (8)	0.0324 (8)	0.0343 (8)	−0.0014 (6)	0.0037 (6)	−0.0014 (6)
C6	0.0487 (10)	0.0363 (9)	0.0379 (9)	0.0024 (8)	0.0057 (8)	−0.0060 (7)
C8	0.0546 (11)	0.0429 (9)	0.0369 (9)	0.0013 (8)	0.0067 (8)	0.0076 (8)
C3	0.0481 (10)	0.0342 (8)	0.0403 (9)	−0.0014 (8)	0.0038 (8)	−0.0056 (7)
C7	0.0585 (12)	0.0504 (10)	0.0303 (9)	0.0041 (9)	0.0051 (8)	−0.0025 (8)
C2	0.0575 (12)	0.0285 (8)	0.0518 (11)	0.0014 (8)	0.0026 (9)	−0.0022 (8)
O7	0.1034 (15)	0.0525 (10)	0.0792 (12)	0.0227 (11)	−0.0132 (11)	−0.0133 (9)
O6	0.0488 (8)	0.0411 (7)	0.0463 (8)	0.0148 (6)	0.0164 (6)	0.0104 (6)
O4	0.0383 (8)	0.0696 (9)	0.0344 (7)	0.0010 (7)	0.0025 (6)	0.0124 (6)
O5	0.0434 (8)	0.0399 (7)	0.0532 (8)	0.0009 (6)	−0.0044 (7)	−0.0073 (6)
O8	0.1058 (18)	0.0780 (13)	0.0705 (12)	−0.0248 (13)	−0.0067 (12)	0.0268 (10)

### Geometric parameters (Å, °)

**Table:**

Mg1—O6	2.0487 (13)	C5—C6	1.391 (2)
Mg1—O6^i^	2.0487 (13)	C6—C7	1.378 (3)
Mg1—O4	2.0570 (13)	C6—H6	0.9300
Mg1—O4^i^	2.0570 (13)	C8—C7	1.373 (3)
Mg1—O5^i^	2.0893 (13)	C8—H8	0.9300
Mg1—O5	2.0893 (13)	C3—C2	1.372 (3)
S1—O3	1.4480 (13)	C3—H3	0.9300
S1—O1	1.4491 (13)	C7—H7	0.9300
S1—O2	1.4504 (13)	C2—H2	0.9300
S1—C1	1.7954 (17)	O7—H7A	0.838 (18)
N1—C1	1.326 (2)	O7—H7B	0.837 (19)
N1—C4	1.346 (2)	O6—H6A	0.85 (3)
N3—C9	1.332 (2)	O6—H6B	0.827 (17)
N3—C8	1.334 (2)	O4—H4B	0.85 (2)
N2—C1	1.322 (2)	O4—H4A	0.827 (17)
N2—C2	1.341 (2)	O5—H5A	0.862 (17)
C4—C3	1.393 (2)	O5—H5B	0.837 (17)
C4—C5	1.480 (2)	O8—H8A	0.85 (3)
C9—C5	1.391 (2)	O8—H8B	0.844 (19)
C9—H9	0.9300		
			
O6—Mg1—O6^i^	180.00 (8)	N3—C9—H9	118.1
O6—Mg1—O4	87.40 (6)	C5—C9—H9	118.1
O6^i^—Mg1—O4	92.60 (6)	C9—C5—C6	117.26 (16)
O6—Mg1—O4^i^	92.60 (6)	C9—C5—C4	119.88 (15)
O6^i^—Mg1—O4^i^	87.40 (6)	C6—C5—C4	122.86 (15)
O4—Mg1—O4^i^	180.0	C7—C6—C5	119.23 (16)
O6—Mg1—O5^i^	92.08 (6)	C7—C6—H6	120.4
O6^i^—Mg1—O5^i^	87.92 (6)	C5—C6—H6	120.4
O4—Mg1—O5^i^	88.83 (6)	N3—C8—C7	122.99 (17)
O4^i^—Mg1—O5^i^	91.17 (6)	N3—C8—H8	118.5
O6—Mg1—O5	87.92 (6)	C7—C8—H8	118.5
O6^i^—Mg1—O5	92.08 (6)	C2—C3—C4	117.84 (16)
O4—Mg1—O5	91.17 (6)	C2—C3—H3	121.1
O4^i^—Mg1—O5	88.83 (6)	C4—C3—H3	121.1
O5^i^—Mg1—O5	180.00 (5)	C8—C7—C6	119.09 (17)
O3—S1—O1	113.91 (8)	C8—C7—H7	120.5
O3—S1—O2	113.33 (8)	C6—C7—H7	120.5
O1—S1—O2	112.78 (8)	N2—C2—C3	123.04 (16)
O3—S1—C1	106.80 (8)	N2—C2—H2	118.5
O1—S1—C1	103.71 (7)	C3—C2—H2	118.5
O2—S1—C1	105.22 (8)	H7A—O7—H7B	106 (4)
C1—N1—C4	116.66 (14)	Mg1—O6—H6A	122.8 (16)
C9—N3—C8	117.68 (15)	Mg1—O6—H6B	125.8 (19)
C1—N2—C2	114.26 (15)	H6A—O6—H6B	108 (2)
N1—C4—C3	119.79 (15)	Mg1—O4—H4B	122.2 (15)
N1—C4—C5	116.42 (14)	Mg1—O4—H4A	118.0 (19)
C3—C4—C5	123.79 (15)	H4B—O4—H4A	114 (2)
N2—C1—N1	128.39 (16)	Mg1—O5—H5A	122.9 (19)
N2—C1—S1	118.06 (13)	Mg1—O5—H5B	116 (2)
N1—C1—S1	113.52 (12)	H5A—O5—H5B	97 (3)
N3—C9—C5	123.75 (16)	H8A—O8—H8B	114 (4)

Symmetry codes: (i) −*x*+1, −*y*+1, −*z*+1.

### Hydrogen-bond geometry (Å, °)

**Table:**

*D*—H···*A*	*D*—H	H···*A*	*D*···*A*	*D*—H···*A*
O4—H4A···O1^ii^	0.83 (2)	1.94 (2)	2.7650 (19)	180 (3)
O4—H4B···O2	0.86 (2)	1.89 (2)	2.7475 (19)	176 (2)
O5—H5B···O3^ii^	0.83 (2)	2.04 (2)	2.8705 (19)	177 (2)
O5—H5A···O8^iii^	0.86 (2)	1.91 (2)	2.755 (3)	167 (3)
O6—H6B···O3^i^	0.83 (2)	2.03 (3)	2.8601 (19)	178 (2)
O6—H6A···N3^iv^	0.85 (3)	1.92 (3)	2.763 (2)	173 (2)
O7—H7B···O3^v^	0.84 (3)	2.51 (3)	3.110 (2)	130 (3)
O7—H7B···N2^v^	0.84 (3)	2.21 (3)	2.984 (2)	154 (3)
O7—H7A···O2	0.84 (3)	2.12 (3)	2.923 (2)	161 (3)
O8—H8B···O1^vi^	0.85 (4)	2.43 (4)	3.064 (2)	132 (4)
O8—H8A···O7^i^	0.85 (3)	1.93 (3)	2.769 (3)	170 (3)

Symmetry codes: (ii) *x*+1, *y*, *z*; (iii) *x*+1/2, −*y*+3/2, *z*+1/2; (i) −*x*+1, −*y*+1, −*z*+1; (iv) *x*+1/2, −*y*+1/2, *z*−1/2; (v) −*x*+1/2, *y*−1/2, −*z*+3/2; (vi) −*x*, −*y*+1, −*z*+1.

## References

[bb1] Bruker (2000). *SADABS*, *SMART *and *SAINT* Bruker AXS Inc., Madison, Wisconsin, USA.

[bb2] Fang, X. B., Dong, H. Z. & Tian, D. B. (2009). *Chin. J. Inorg. Chem.* **25**, 47–53.

[bb3] Kimura, K., Kimura, T., Kinoshita, I., Nakashima, N., Kitano, K., Nishioka, T. & Isobe, K. (1999). *Chem. Commun.* pp. 497–498.

[bb4] Lobana, T. S., Kinoshita, I., Kimura, K., Nishioka, T., Shiomi, D. & Isobe, K. (2004). *Eur. J. Inorg. Chem.* pp. 356–367.

[bb5] Sheldrick, G. M. (2008). *Acta Cryst.* A**64**, 112–122. 10.1107/S0108767307043930 18156677

[bb6] Zhu, H. B., Dong, H. Z., Huang, W. & Gou, S. H. (2007). *J. Mol. Struct.* **831**, 55–60.

